# Cancer cell-binding peptide fused Fc domain activates immune effector cells and blocks tumor growth

**DOI:** 10.18632/oncotarget.12445

**Published:** 2016-10-04

**Authors:** Anne Mobergslien, Qian Peng, Vlada Vasovic, Mouldy Sioud

**Affiliations:** ^1^ Department of Cancer Immunology, Institute for Cancer Research, Oslo University Hospital-Radiumhospitalet, N-0310 Oslo, Norway; ^2^ Department of Pathology, Institute for Cancer Research, Oslo University Hospital-Radiumhospitalet, N-0310 Oslo, Norway

**Keywords:** Fc-fusion proteins, innate effector cells, NK cells, immunotherapy, immunostimulation

## Abstract

Therapeutic strategies aiming at mobilizing immune effector cells to kill tumor cells independent of tumor mutational load and MHC expression status are expected to benefit cancer patients. Recently, we engineered various peptide-Fc fusion proteins for directing Fcg receptor-bearing immune cells toward tumor cells. Here, we investigated the immunostimulatory and anti-tumor effects of one of the engineered Fc fusion proteins (WN-Fc). In contrast to the Fc control, soluble WN-Fc-1 fusion protein activated innate immune cells (e.g. monocytes, macrophages, dendritic cells, NK cells), resulting in cytokine production and surface display of the lytic granule marker CD107a on NK cells. An engineered Fc-fusion variant carrying two peptide sequences (WN-Fc-2) also activated immune cells and bound to various cancer cell types with high affinity, including the murine 4T1 breast carcinoma cells. When injected into 4T1 tumor-bearing BALB/c mice, both peptide-Fc fusions accumulated in tumor tissues as compared to other organs such as the lungs. Moreover, treatment of 4T1 tumor-bearing BALB/c mice by means of two intravenous injections of the WN-Fc fusion proteins inhibited tumor growth with WN-Fc-2 being more effective than WN-Fc-1. Treatment resulted in tumor infiltration by T cells and NK cells. These new engineered WN-Fc fusion proteins may be a promising alternative to existing immunotherapies for cancer.

## INTRODUCTION

In spite of significant progress in recent years towards the development of new targeted therapies, cancer is still a largely unmet medical need [1]. Among the new therapeutics, checkpoints blocking antibodies, such as anti-CTLA-4 and anti-PD1, represent a therapeutic strategy that has shown great promise in cancer [2]. However, checkpoint inhibitors work best in tumors with high mutational load (neoantigens) such as melanoma, but are less successful in many solid tumors and B cell malignancies [3, 4, 5]. Moreover, most tumors escape traditional T-cell killing via alteration of their antigen processing machinery and/or down-regulation of MHC class I expression, rendering the neoantigens undetectable by the immune system [6]. In contrast to checkpoint inhibitors, the clinical efficacy of several monoclonal antibodies (mAbs) such as retixumab, herceptin, and cetixumab, is largely dependent on the patient's innate immune effector cells [7]. Indeed, the engagement of the antibody Fc domain with the Fcγ receptors expressed by innate immune cells such as natural killer (NK) cells and macrophages triggers antibody-dependent cellular cytotoxicity (ADCC) and phagocytosis of tumor cells [9, 10]. The contribution of ADCC to antibody efficacy in patients is also supported by studies demonstrating associations between therapeutic efficacy and the expression of certain Fcγ receptor allotypes [11]. FcγR activation can be stimulatory or inhibitory to effector cells, depending on which receptor is triggered and which cells are activated [12]. With respect to cancer immunotherapy, NK cells are central in that they express only the activating FcγRIIIa receptor (CD16) and no inhibitory antibody receptors underscoring a major role in promoting tumor cell killing [13].

Although Ab therapy has led to important clinical advances, most of the used Abs have targeted surface antigens whose repertoire on solid tumors is limited [11]. Owing to their full-size, Abs are also impeded from entering solid tumors, calling for the development of new targeted therapies [14]. To increase the spectrum of tumor antigens that can be targeted by Abs in solid tumors, we have explored the use of cancer cell-binding peptides selected from peptide phage libraries as a targeting moiety [15]. By fusing such peptides to human IgG-1 Fc domain, we have married the targeting specificities of the peptides with the effector function of Abs such as the induction of ADCC. The engineered peptide-Fc fusion proteins, particularly the WN-Fc variant, bound to various cancer cell types. Given the involvement of innate immune cells in antibody clinical efficacy and the binding of the engineered peptide Fc fusions to cancer cells, here we have investigated whether soluble WN-Fc fusion proteins could function as an adjuvant and inhibit tumor growth in mice.

## RESULTS

### Engineering WN peptide-Fc fusion proteins

**Figure 1:**
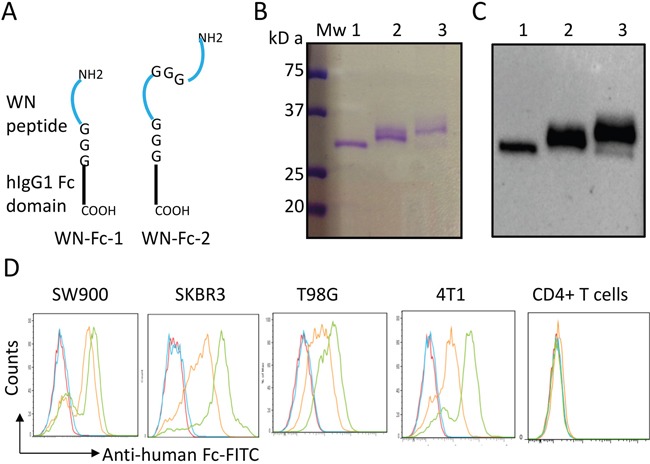
Characterization of the peptide-Fc fusion proteins **A.** Schematic representation of WN-Fc-1 and WN-Fc-2 constructs. **B.** Analysis of purified Fc fusion proteins by SDS-PAGE followed by Coomassie staining. **C.** Western blot analysis with an anti-human Fc monoclonal antibody. Lanes 1 to 3 correspond to Fc control, WN-Fc-1, and WN-Fc-2 fusion proteins, respectively. **D.** Binding of Fc control, WN-Fc-1, and WN-Fc-2 to cancer cell lines and human CD4+ T cells. The cells were stained with the recombinant proteins (5μg/ml) and then analyzed by flow cytometry. Blue, orange and green histograms represent cells stained with Fc control, WN-Fc-1, or WN-Fc-2, respectively. Red histograms = cells stained with only FITC-conjugated anti-human Fc antibody. The data are from one single experiment and are representative of five independent experiments.

We first wanted to improve the binding affinity of the WN-Fc fusion protein to cancer cells by fusing two copies of the targeting WN peptide (WNLPWYYSVSPT) to a single human IgG1 Fc domain as illustrated in Figure 1A. Recombinant peptide-Fc fusion proteins were expressed in HEK293 cells and then purified on protein G chromatography. The purity of the engineered proteins was higher than 98% as revealed by SDS-PAGE and Western blots (Figure 1B and 1C). As a result of glycosylation, the apparent molecular mass of recombinant human Fc is around 32 kDa in SDS-PAGE under reducing conditions. Under our experimental conditions, both WN-Fc-1 and WN-Fc-2 ran as slightly diffuse bands; reflecting structure-related effect.

We next assessed the ability of the engineered proteins to bind human lung (SW900), breast (SKBR3), and glioma (T98G) cancer cell lines (Figure 1D). In contrast to the Fc control (blue histograms), WN-Fc-1 displayed a strong binding to all three tested cell lines (orange histograms). The expression of two peptide sequences in a single Fc domain (WN-Fc-2) increased the binding (green histograms). As shown in Figure 1D, WN-Fc-1 and WN-Fc-2 fusion proteins bound to 4T1 murine mammary carcinoma cell line, while no significant binding was seen with human peripheral blood CD4+ T lymphocytes. Under our experimental conditions, The WN-Fc-2 bound to SW900, SKBR3, T98G, and 4T1 cells with apparent affinities of 15 ± 3 nM, 7 ± 2 nM, 20 ± 5 nM, and 8 ± 2 nM, respectively.

### Soluble WN-peptide-Fc fusion proteins activate immune cells

**Figure 2:**
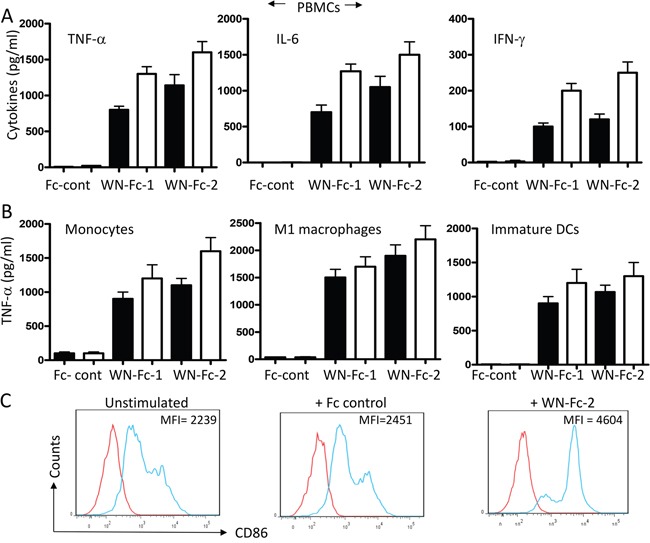
Activation of blood cells in response to soluble WN-Fc-1 and WN-Fc-2 **A.** PBMCs were incubated for 16 h in X-VIVO 15 medium alone or medium supplemented with WN-Fc-1, WN-Fc-2, or Fc control (5 or 10 μg/ml, black and white columns, respectively). Culture supernatants were harvested and analyzed for TNF-α and IFN-γ contents by ELISA. **B.** Similarly, monocytes, M1 macrophages, and immature DC were stimulated with the test molecules as described in A and TNF-α contents in culture supernatants were determined by ELISA. The results in A and B are represented as means ± SD from triplicate determinations and are representative of at least five independent experiments using NK cells from different donors. **C.** Expression of CD86 co-stimulatory molecule. Immature DCs were stimulated with Fc control or WN-Fc-2 (10 μg/ml each) for 48 h and then the CD86 expression was analyzed by flow cytometry. The results are representative of four independent experiments.

Engineering anti-tumor Fc-fusion proteins for optimal activity requires a clear understanding of their interactions with immune effector cells. Therefore, we assessed the effects of the engineered proteins on cytokine production by freshly isolated peripheral blood mononuclear cells (PBMCs). Following 16 h stimulation of PBMCs with WN-Fc-1, WN-Fc-2 or Fc control, culture supernatants were harvested and cytokine contents were measure by ELISA (Figure 2A). Cells stimulated with WN-Fc-1 or WN-Fc-2 secreted significant levels of TNF-α, IL-6 and INF-γ, whereas those stimulated with Fc control did not secrete cytokines when compared to unstimulated cells. Similarly, treatment of blood monocytes, M1 macrophages and immature DCs with WN-Fc-1 and WN-Fc-2 induced TNF-α expression (Figure 2B). Again, stimulation with the Fc control had no effect on cytokine production. M2 macrophages also responded to WN-Fc fusion proteins (data not shown). Immature DCs exposed to WN-Fc-2 fusion protein upregulated the expression of costimulatory molecules such as CD86 (Figure 2C). A significant increase in mean fluorescence intensity is evident when compared to the Fc control-treated cells (4604 versus 2451, *P*<0.001). Collectively, these results indicate that the WN-Fc fusion proteins could be used as adjuvant to activate immune effector cells such as macrophages and DCs, an important antigen presenting cells for promoting immunity against tumor cells.

### WN-Fc fusion proteins efficiently activate NK cells

**Figure 3:**
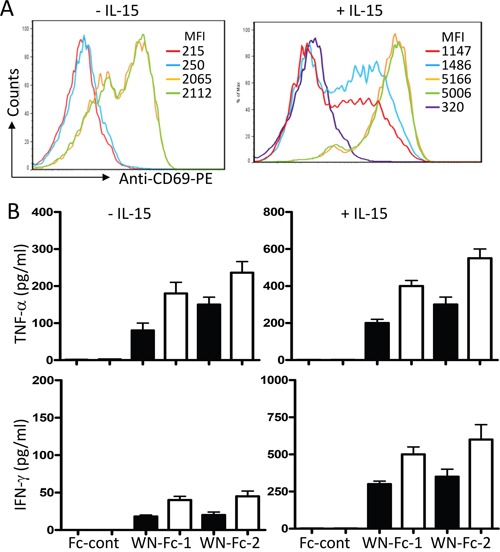
Activation of NK cells in response to WN-Fc-1 and WN-Fc-2 **A.** NK cells were stimulated with WN-Fc-1, WN-Fc-2, or Fc control (10 μg/ml each) for 16 h in X-VIVO 15 supplemented (+) or not (-) with IL-15. Thereafter, the cells were stained for CD69 expression and analyzed by flow cytometry. The data are representative of at least five independent experiments. MFI= mean fluorescence intensity. **B.** As in A, NK cells were stimulated with the test molecules (5 or 10 μg/ml, black and white columns, respectively) for 16 h and then secreted TNF-α and IFN-γ were measured by ELISA. The results are represented as means ± SD from triplicate determinations and are representative of five independent experiments using NK cells from different donors.

Given the central role played by NK cells in cancer immunotherapy [9], we next evaluated the relative ability of soluble WN-Fc-1 and WN-Fc-2 to stimulate NK cell FcγRIIIa receptor. Freshly isolated blood NK cells were stimulated for 16 h with WN-Fc-1 or WN-Fc-2 in the presence or absence of IL-15 (10 ng/ml). As illustrated in Figure 3A, WN-Fc-1 and WN-Fc-2 activated resting NK cells as revealed by the up-regulation of CD69 activation marker (orange and green histograms, respectively). Under the same experimental conditions, soluble Fc control did not induce CD69 expression (blue histogram). Additional exposure of NK cells to IL-15 generally increased their reactivity without altering the differential effects of WN-Fc-1 and WN-Fc-2 over the Fc control (Figure 3A, a representative example). We also measured TNF-α and INF-γ production in response to soluble WN-Fc-1 and WN-Fc-2 (Figure 3B). NK cells stimulated for 16 h with WN-Fc-1 or WN-Fc-2 secreted TNF-α and IFN-γ, whereas those stimulated with Fc control did not. Similarly, cytokine production was enhanced when NK cells were co-stimulated with IL-15, which is an important regulator of NK cell development, homeostasis and activation [17].

### WN-Fc fusion proteins induce NK cell degranulation

**Figure 4:**
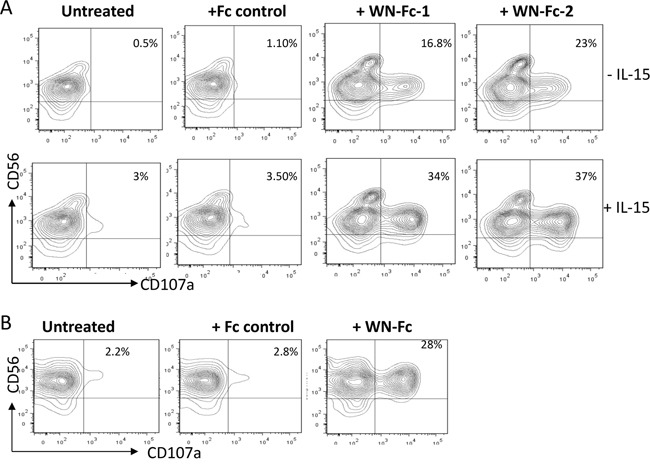
NK cell degranulation **A.** Surface expression of CD107a by NK cells. Resting or IL-15-activated NK cells were stimulated with the indicated Fc fusion proteins (10 μg/ml each) and then analyzed for CD107a surface expression after 5 h incubation at 37°C in X-VIVO 15 medium. **B.** WN-Fc-coated SKBR3 activated NK cells. SKBR3 cells were incubated with WN-Fc-2 or Fc control for 30 min at room temperature. Thereafter, the cells were washed to remove unbound molecules and then incubated with NK cells for 5 h at 37°C and processed as in A. The data in A and B are from one single experiment and are representative of five independent experiments using NK cells from different donors. The numbers in dot plots represent the percentage of CD107a-positive NK cells.

Recently, CD107a (also known as lysosomal-associated membrane protein-1, LAMP-1) expression on the cell surface has been described as a marker of NK cells and cytotoxic T cell degranulation [19, 20]. Such surface display also correlated with perforin secretion and destruction of tumor cells. Therefore, we measured the surface levels of CD107a in response to soluble WN-Fc-1 and WN-Fc-2 (Figure 4A). The percentage of CD107a positive cells was increased after 5 h stimulation. In the case of WN-Fc-2, CD107a expression on the surface of resting NK cells increased 21-fold, resulting in 23% of CD56 positive cells expressing CD107a. Pre-activation of NK cells with IL-15 enhanced WN-Fc-1 and WN-Fc-2 specific effects on NK cell degranulation (37% versus 23% and 34% versus 16.8%, respectively, *P*< 0.01, n=5). Similarly, WN-Fc-2-coated SKBR3 cells activated NK cells, leading to surface display of CD107a (Figure 4B). In these experiments, tumor cells were incubated with WN-Fc-2 for 30 min and then unbound WN-Fc molecules were removed by washing prior to co-stimulation with NK cells.

### Induction of ADCC by WN-Fc-2 fusion protein

**Figure 5:**
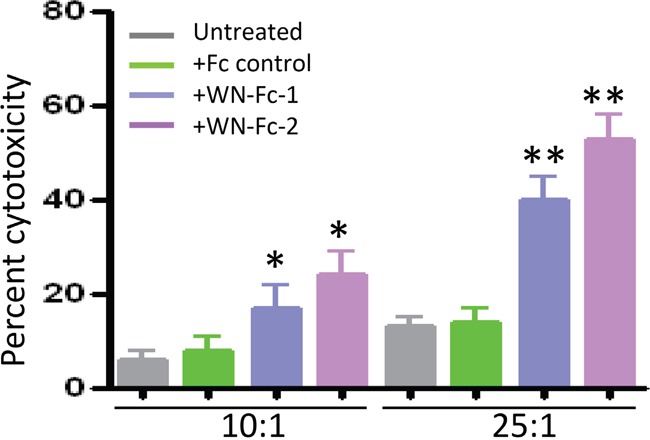
Induction of ADCC by WN-Fc fusion proteins ADCC as measured by LDH release. SKBR3 target cells were pre-incubated with WN-Fc-1, WN-Fc-2, or Fc control for 30 min in X-VIVO medium. Thereafter, freshly isolated NK cells were added to the cells at ratio 12.5:1 or 25:1 effector to target cells. After 18 h incubation at 37°C, culture supernatants were collected and analyzed for LDH. The results are represented as means ± SD from triplicate determinations and are representative of four independent experiments using NK cells from different donors. Statistically significant differences between NW-Fc-2 treated cells and Fc control-treated cells are indicated by asterisks. *P<0.05, **P<0.02.

The potency of WN-Fc-2 to induce ADCC was subsequently evaluated using SKBR3 as target (T) cells and freshly isolated NK cells as effector (E) cells based on LDH release upon cell lysis [21]. As shown in Figure 5, Both WN-Fc-1 and WN-Fc-2 induced a significant ADCC activity against SKBR3 (40% ± 5% and 53% ± 8%, respectively, at E/T ratio 25:1) in comparison with cells incubated with the Fc control (13% ± 3%, at E/T ratio 25:1) (*P*<0.02, n=5). Thus, WN-Fc fusion proteins can function as immunostimulator and ADCC inducers. Again WN-Fc-2 showed more cytotoxic activity than WN-Fc-1.

### Targeting 4T1 cells *in vitro* and *in vivo*

**Figure 6:**
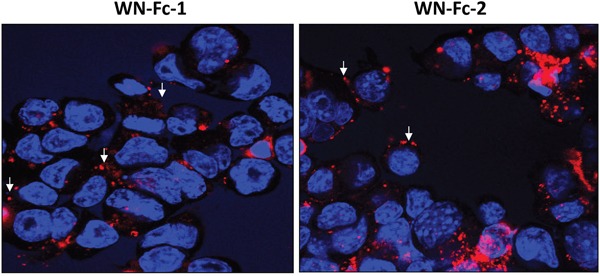
Binding and cellular uptake of WN-Fc fusion proteins 4T1 cells growing in Lab-Tek chamber slides were incubated with rhodamine-conjugated WN-Fc fusion proteins, washed, fixed with paraformaldehyde, and then analyzed by confocal microscopy to check for surface binding and internalization. Nuclei were visualized with Hoeschst 33342 staining (blue). Representative images are shown (original magnification x 60). The arrows indicate membrane staining.

Binding of the WN peptide to its receptor has been shown to promote receptor internalization [22]. Therefore, we investigated whether WN-Fc fusion proteins are also internalized by 4T1 cells. Cells grown in tissue culture slides were incubated with rhodamine conjugated WN-Fc fusion proteins at 4°C. After washing, the cells were incubated at 37°C for 2 h to promote internalization. Both cell surface and intracellular staining were detected, indicating that cell-bound WN-Fc fusion proteins were being internalized as well (Figure 6). Similarly, antibodies targeting epidermal growth factor receptor, such as Cetuximab which is approved for clinical use, also induce receptor internalization and ADCC [23].

**Figure 7:**
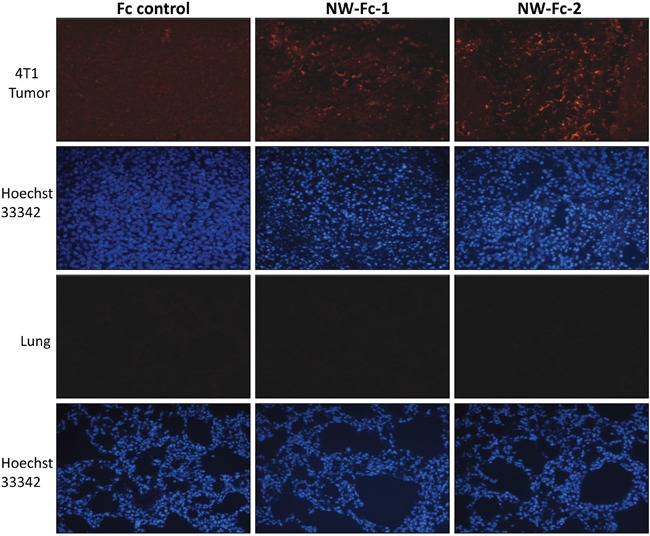
Fluorescence microscopy analysis of tumors and lung tissues Rhodamine-conjugated NW-Fc fusion proteins or Fc control (200 μg each) was intravenously injected into the tail vein of mice bearing subcutaneous 4T1 tumors. After 18 h, tumors and others organs were removed, fixed with paraformaldehyde and prepared for frozen sections. Tissue sections were incubated with Hoechst 33342 for nuclei staining (blue) and mounted using Dako fluorescent mounting medium before analysis by an epifluorescence microscope. Representative images from each group are shown (original magnification x 10).

To determine whether WN-Fc fusion proteins could target tumor cells *in-vivo*, rhodamine conjugated WN-Fc fusion proteins were injected i.v. into 4T1 tumor-bearing BALB/c mice, before removing the tumors 18 h later to assess 4T1 tumor and normal organs for fluorescence (Figure 7, representative examples). As opposed to lung tissues, the data confirm the accumulation of WN-Fc fusion proteins in tumor tissues. Additionally, no significant fluorescence was detected in heart and kidney tissues (data not shown).

### Inhibition of tumor growth by the WN-Fc-2 fusion protein

**Figure 8:**
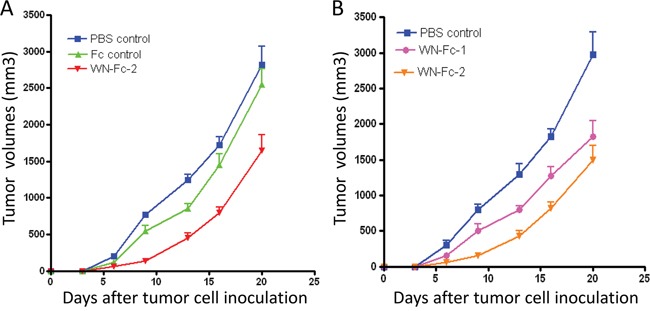
Inhibition of tumor growth in BALB/c mice **A.** 4T1 s.c. tumor-bearing mice were treated on day 3 and 7 (i.v. injection) with PBS, Fc control or WN-Fc-2 (100 μg/200μl PBS per mouse). Tumor dimensions were measured and then volumes were calculated. Each point represents the mean of 7 determinations (n=7) per group; bars = SD. **B.** Effects of WN-Fc-1 and WN-Fc-2 on tumor growth. Experimental conditions are as in A.

The Fc domain of human IgG1 seems to interact equally with murine and human effector cells such as NK cells [24]. Hence, mouse models are suitable for evaluating the Fc-mediated effect of human IgG1 mAb [25]. Given the binding of WN-Fc-2 to 4T1 murine mammary carcinoma cells (Figure 1D), we thus investigated its therapeutic potency using the 4T1 tumor model in syngeneic female BALB/c mice. The Fc control and WN-Fc-2 were administrated intravenously (100 μg/mouse/injection) at day 2 and 7 after subcutaneous tumor cell inoculation and tumor growth was monitored until day 20. Tumors were also harvested and weighted at day 20. As shown in Figure 8A, the average tumor volumes of mice receiving WN-Fc-2 were significantly smaller than those of the PBS- or Fc-control-treated mice (*P*<0.005 for WN-Fc-2 treatment versus PBS-treatment and *P*<0.01 for WN-Fc-2 treatment versus Fc control at day 20). When compared to both untreated and mice treated with the Fc control, the WN-Fc-2 significantly inhibited tumor growth at all time points. At day 20, WN-Fc-2 treatment reduced the mean tumor weight from 1.8 to 1.06 g (*P*<0.003, n =7 mice per group**).** We have also compared the anti-tumor potency of WN-Fc-1 and WN-Fc-2 (Figure 8B). Treatment with WN-Fc-1 showed a significant anti-tumor effect when compared to untreated animals (*P*<0.01, n=7 mice per group). Notably, WN-Fc-2 showed superior *in vivo* efficacy than WN-Fc-1 at the same concentration.

### WN-Fc treatment enhances immune cell recruitment into tumors

**Figure 9:**
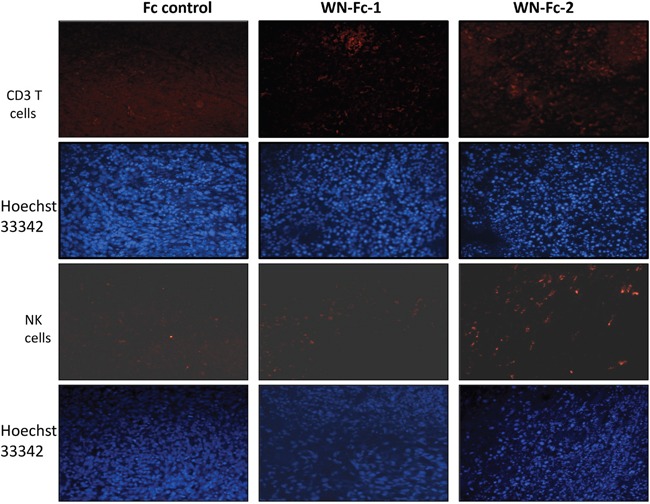
Analysis of T cells and NK cells infiltration into tumor tissues Tumors were removed on day 14 after treatment and frozen sections were stained with phycoerytrin-conjugated mouse anti-CD3 or phycoerytrin-conjugated NKp46 monoclonal antibodies. Representative immunofluorescence microscopy images showing the presence of CD3+ T cells and NK cells in WN- Fc treated animals. Blue, Hoeschst 33342-stained nuclei.

Increase lymphocyte infiltration within tumors has been observed in several tumors subsequent to therapy with Abs or with conventional therapies such as chemotherapy [26]. Since WN-Fc fusions inhibited tumor growth, we next assessed whether they would enhance immune cell infiltration into tumors. Immunohistochemical staining revealed an increase in CD3+ T cells and NK cell infiltration in the tumors of WN-Fc-treated mice when compared to tumors-derived from mice treated with the Fc control (Figure 9, representative examples). WN-Fc-2 treatment seems to recruit more lymphocytes into tumors than that of WN-Fc-1. Regardless of the difference, the data support the use of WN-Fc fusion proteins to mobilize immune cells into tumor tissues.

## DISCUSSION

**Figure 10:**
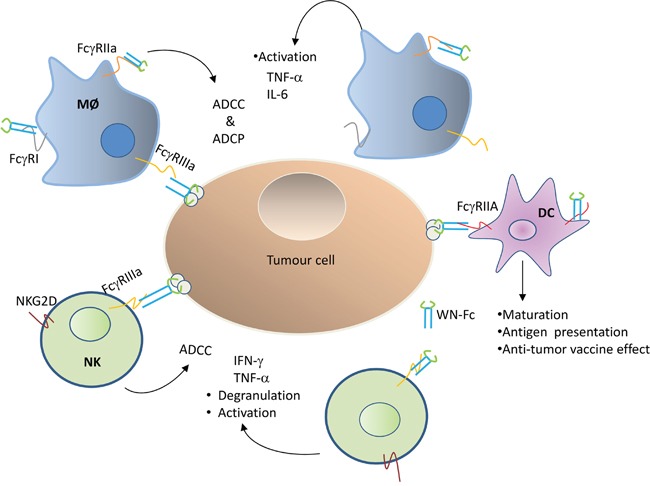
Schematic diagram showing Fcγ receptor interaction with WN-Fc fusion proteins Both soluble and NW-Fc-coated tumor cells activate innate immune cells such as NK cells, macrophages (MØ), and dendritic cells (DC) via different types of activating Fc-γ receptors: FcγR1 (CD64), FcγRIIa (CD32a), FcγRIIIa (CD16a). ADCC = antibody-dependent cellular cytotoxicity, ADCP = antibody-dependent cellular phagocytose.

Fc-based fusion proteins, in which the Fc domain of an antibody of the IgG isotype is fused to a different protein, have merged as an important class of new pharmaceuticals [27]. To date, most of the engineered Fc fusion proteins either work as antagonists to block receptor-ligand interactions or as agonists to stimulate the receptor function [27]. In this study, we have shown that WN-Fc fusion proteins can serve as a potent activator for immune effector cells such as NK cells, monocytes, and DCs (Figure 10). Importantly, treatment of 4T1 tumor-bearing mice with WN-Fc- fusion proteins inhibited tumor growth, providing support for the rational use of WN-Fc fusion proteins as adjuvant and tumor cell killers.

With respect to cancer immunotherapy, the Fc domain of Ab mediates cellular cytotoxic functions through its interactions with the Fcγ receptors (activating receptors FcγRI, FcγRIIa and FcγRIIIa; inhibitory receptor FcγRIIb). Moreover, cytokine production by innate immune cells seems to be important for clinical responses to therapeutic Abs [28]. Indeed, IFN-γ and TNF-α are known to enhance NK cytotoxicity and macrophage phagocytosis of tumor cells [10]. Hence, the observation that both soluble and WN-Fc-coated tumor cells can activate innate immune cells is interesting. Given that soluble Fc control did not trigger cytokine production, it seems that the nature of the peptide sequence fused to the Fc domain clearly affects the effector function of the engineered proteins. Based on the present data, we thus propose that the structure formed by WN-Fc-1 and WN-Fc-2 fusion proteins may facilitate their interaction with the Fcγ receptors expressed by innate immune cells. Even in the presence of 0.1% SDS and under reducing conditions, WN-Fc fusion proteins retained some conformational behavior (see Figure 1B and 1C). It should be noted that free WN peptide has no effect on innate immune cells, again arguing for WN-Fc structure-related effect. Moreover, none of the other tested soluble peptide Fc-fusions activated innate immune cells (data not shown), thus the observed adjuvant effect seems to be specific for WN-Fc fusion proteins.

As the immune response is a major determinant of therapeutic Ab efficacy in cancer patients, the opportunity now exists to combine Ab therapy with immunostimulators to enhance objective clinical responses [16]. Various strategies including stimulating the innate response and blocking inhibitory signals are being explored in combination with Abs [17, 18]. With respect to cytokines, IL-15 has many activating and homeostatic functions on lymphocytes, and functions at different stages of the immune response by expanding and activating NK cells [29]. Ajuvants such as CpG oligodeoxynucleotides (ODN) and RNA oligonucleotides, ligands for Toll like receptors (TLRs) 9 and 7/8, respectively, have been used in cancer immunotherapy [30, 32]. In this respect, Moga and colleagues showed that IL-15 or CpG ODN can enhance rituximab-induced ADCC against B-cell lymphoma [32]. TLR ligands also activated NK cells and enhanced trastuzumab- and rituximab-induced ADCC *in-vitro* and *in-vivo* [33–35]. Similarly, immunomodulatory drugs such as lenalidomide enhanced rituximab-induced ADCC against lymphoma cell lines and primary B-cell chronic lymphocytic leukemia cells via NK cells and monocyte activation [36]. Together, these observations are consistent with the notion that immune-enhancing properties of various agents can improve Ab therapeutic efficacy.

In principle, an ideal cancer therapeutic protein should not only engage Fcγ RIIIA on NK cells, but also activate other innate cells, particularly macrophages and DCs. By undergoing activation and maturation, DCs are expected to present tumor antigens/neoantigens to T cells. To prevent an excessive and prolonged response that could result in tissue destruction or autoimmunity, activated immune cells are controlled via the expression of co-inhibitory molecules [2, 37]. Activated T cells, macrophages, and NK cells express multiple co-inhibitory receptors and intracellular factors. While these immune checkpoint factors control the normal function of the immune system, when expressed during immunotherapy, they represent a significant barrier for the induction of a strong and sustained anti-tumor immune response, which is required for tumor cell destruction. In addition to the adjuvant effect, signaling via TLR or IL15 receptor also induces the expression of suppressor of cytokine signalling (SOCS) proteins, particularly SOCS-1, that attenuate or terminate intracellular signalling [38, 39]. Therefore, blocking of SOCS-1 expression in adoptive transferred NK cells or T cells could further enhance anti-tumour potency of this form of therapy [40].

Immune-compromised models such as human cell line derived xenografts are useful for assessing the preclinical responses to conventional cancer treatments. However, the use of immune-competent models is essential when studying the effects of immunotherapeutics. The designed WN-Fc fusions proved to be effective in inhibiting 4T1 tumor growth in immune-competent BALB/c mice. The selected therapeutic dose (around 5 mg/kg) is based on preclinical studies with Abs [23, 27]. Under our experimental conditions, no side effects such as loss of weight and decrease in spontaneous *movement* were seen in mice exposed to 5, 10, and or 15 mg/kg dose. Notably, WN-Fc treatment enhanced CD3 T cells and NK cells infiltration into tumor tissues. Subsequent to activation, NK cells mediate their effector functions through perforin and granzyme release as well as secretion of cytokines such as IFN-γ and TNF-α [10]. Similarly, treatment with either CTLA-4 or PD1 blocking antibodies increased lymphocyte infiltration in tumor tissues [41, 42].

Although the identified WN peptide can be used to direct innate immune cells against tumor cells without knowledge of its cellular receptor, moving this peptide towards clinical use will be facilitated by the identification of its binding receptor. We have performed a series of experiments (e.g. affinity capture, immunoprecipitation) to identify the WN-peptide receptor and the data suggest the involvement of several glycoproteins. Hence, the binding may arise from the formation of a native conformational epitope involving the interaction of more than one protein. Such interaction is more likely to be lost upon the preparation of membrane proteins. While further work is needed before patients with cancer can be treated with the WN-Fc fusion proteins, the results identify an unexpected effect of soluble WN-Fc fusions on innate immune cells and support efforts to evaluate the potential of the engineered peptide- Fc fusion proteins for use in cancer immunotherapy.

## MATERIALS AND METHODS

### Cells and antibodies

Human cancer cell lines SKBR3 (breast), SW900 (lung), T98G (glioma), and murine 4T1 breast carcinoma cell line were obtained from the American Type Culture Collection (ATCC, Rockville, MA). Human peripheral blood mononuclear cells (PBMCs) were isolated from buffy coats by lymphoprep density gradient centrifugation. Monocytes were enriched from PBMCs using plastic adherence and purification was verified by phenotypic analysis of the surface marker CD14+. To generate M1 and M2 macrophages, monocytes were cultured for 6 days in RPMI-1640 medium in the presence of 100 ng/ml GM-CSF (M1) or 100 ng/ml M-CSF (M2) as described by Rey-Giraud et al. [43]. NK cells were purified from PBMCs using NK cell isolation kit and auto MAC Pro Separator according to the manufacturer's instructions (Miltenyi Biotec, GmbH). Purification was verified by phenotypic analysis of the surface marker CD56. Cells were cultured in RPMI-1640 or DMEM supplemented with 10% heat-inactivated fetal calf serum and antibiotics. Cell stimulation with WN-Fc-1 or WN-Fc-2 was performed in X-VIVO 15 medium (Lonza, Basel, Switzerland). Approval for obtaining buffy coats from normal volunteers was granted by Oslo University Hospital Ethics Committee. Anti-CD56 and Anti-CD107a antibodies were purchased from BD Pharmingen (San Diego, CA, USA). FITC-conjugated anti-human Fc was purchased from Sigma (St. Louis MO USA). PE-conjugated anti-CD14, anti-CD80, anti-CD86, and anti-CD69 monoclonal antibodies were purchased from Dako (Glostrup, Denmark). PE-conjugated mouse anti-CD3 and anti-NKp46 were purchased from Biolegend.

### Cytokine production and NK cell degranulation

PBMCs, monocytes, M1 and M2 macrophages, immature DCs and NK cells were cultured for 16 h in X-VIVO 15 medium supplemented with WN-Fc-1, WN-Fc-2, or Fc control (5 or 10 μg/ml each). Subsequently, culture supernatants were collected and cytokine contents were measured by ELISA. CD69 expression as marker for NK activation was analyzed by FACS subsequent to 16 h stimulation time. To measure surface expression of CD107a/LAMP-1, a surrogate marker for NK cell degranulation, NK cells (5×10^4^ in 200 μl X-VIVO medium) were stimulated with WN-Fc-1, WN-Fc-2, or Fc control (10 μg/ml). During stimulation, PE-Cy5 conjugated anti-CD107a (2 μl/well) and monensin (0.2 μl/well) were added to the cells. After 5 h incubation at 37°C, the cells were harvested, washed, stained with FITC conjugated anti-CD56 and then analyzed by FACSCanto II flow cytometer (BD Biosciences, San Jose, CA, USA). All flow data were analyzed by flowJo software.

### Oligonucleotides, cloning, and expression

The following overlapping DNA oligonucleotides encoding peptides were made and HPLC purified by Eurofins Genomics (Ebersberg, Germany).

WN-Fc-1 peptide (WNLPWYYSVSPT) 5′-aattcgtggaatcttccttggtattatagcgtcagtcctacgggtggaggca-3′ 5′-gatctgcctccacccgtaggactgacgctataataccaaggaagattccacg-3′ WN-Fc-2 peptide (WNLPWYYSVSPT**GGG**WNLPWYYSVSPT) 5′-aattcgtggaatcttccttggtattatagcgtcagtcctacgggtggaggctggaa tcttccttggtattatagcgtcagtcctacgggtggaggca-3′ 5′-gatctgcctc cacccgtaggactgacgctataataccaaggaagattccagcctccacccgtagga ctgacgctataataccaaggaagattccacg-3′ Fc control peptide (ISAMVRS) 5′-aattcgatatcggccatggtta-3′ 5′-aattcgatatcggcc atggtta-3′

DNA oligonucleotides were annealed together to form a double stranded sequence with overhanging bases for *EcoR1* and *BglII* restriction sites and then cloned into *EcoR1-BglII*-cleaved pFuse-hIgG1-Fc2 vector in frame with IL-2 signal sequence and the Fc portion of human IgG1 (In vivoGen, San Diego, CA, USA). Recombinant peptide Fc fusion proteins were produced by transient transfection of the plasmids into HEK293T cells and purified by protein G chromatography. Protein purity was determined by electrophoresis on 10% sodium dodecyl sulfate (SDS) polyacrylamide gel and positive fractions were collected, pH adjusted to 7.5 and then stored at −80°C until use.

### Flow cytometry

The binding of the WN-Fc fusion proteins to cancer cells was analyzed by flow cytometry. In brief, aliquots of cells (10^5^) were divided into conical 96-well micro-plate, washed with PBS buffer containing 1% FCS, and then incubated with the affinity-purified peptide-Fc fusions for 30 min on ice. After washing, cells were incubated with FITC-conjugated anti-human Fc for 30 min on ice and then analyzed by flow cytometry.

### Antibody dependent cellular cytotoxicity assay (ADCC)

ADCC was conducted using the lactate dehydrogenase (LDH) cytotoxicity kit in accordance with manufacturer's instructions (Promega, Madison, WI, USA). Cancer cells pre-treated with the peptide-Fc fusions (10 μg/ml) were incubated with IL-15-activated NK cells for 18 h at 37°C. NK cells were prepared from human peripheral blood mononuclear cells. After incubation, culture supernatants were transferred to a 96-well plate to determine the amount of LDH released. Percentage of cell lysis in the cytotoxicity assays was calculated as (experimental release – background release/maximum release – background release) x 100. The effector-to-target ratio 12:1 and 25:1 was determined from pilot experiments.

### Analysis of NW-Fc fusion protein binding to 4T1 cells by fluorescence microscopy

4T1 cells were cultured in Lab-Tek chamber slides (Nalge Nunc International, Naperville, USA) for 24 h in X-VIVO 15 medium. Then the medium was replaced with fresh medium and the cells were incubated with Rhodamine conjugated WN-Fc fusion proteins (10 μg/ml) for 30 min at 4°C. Subsequently, the cells were washed and incubated at 37°C for 2 h to allow internalization, followed by 5 min incubation with Hoechst 33342. The cells were washed twice with PBS and fixed with 4% paraformaldehyde for 15 min at 4°C. After washing, slides were covered with Dako cytomation fluorescent mounting medium and then images were taken with a confocal microscope (Zeiss LSM 510, Olympus, Tokyo, Japan).

### Treatment of subcutaneous 4T1 metastatic breast cancer

Female BALB/c mice were purchased from Harlan (Boxmeer, The Netherlands) and housed according to the experimental animal guidelines. They were acclimated to the new environment for at least one week after arrival before being used in experiments. The murine 4T1 breast carcinoma cell line was grown in monolayer culture and harvested by trypsinization prior to cell inoculation. BALB/c female mice 5 to 7 weeks of age were injected subcutaneously with 25 x10^5^ tumor cells and mice were randomized into three different treatment groups (7 mice per group). Test molecules were administrated intravenously at day 2 and 7 after tumor inoculation (100 μg/mouse/injection). Animals were assessed daily for clinical symptoms and adverse effects. Tumor growth was monitored until sacrifice at day 20. The same experimental conditions were used for the next set of experiments. All experiments involving mice were approved by the National Animal Research Authority (Oslo, Norway) and performed according to international standards.

### Analysis of the tumor-associated WN-Fc fusion proteins by fluorescence microscopy

Around two weeks after tumor cell inoculation, rhodamine conjugated WN-Fc fusion proteins were injected intravenously into separate animals using 200 μg of the conjugate in 100 μl of physiological saline. Mice were sacrificed 18 h after injection and tumors and other organs (lungs, kidneys and heart) were removed and incubated overnight in 4% paraformaldehyde solution and then embedded in optimal cutting temperature medium (OCT). Tissue sections were incubated with Hoechst 33342 for nuclei staining. Thereafter, the slides were covered with Dako cytomation fluorescent mounting medium before examination under an epifluorescence microscope (Leica DM RHC, Leica Microscopy As, Oslo).

### Analysis of tumour infiltrating lymphocytes

Detection of T cells and NK cells in tumors and normal organ tissues was conducted according to standard immunohistochemical methods using 5-μm sections of OCT-embedded tumors or organ tissues fixed with paraformaldehyde. For detection of T cells and NK cells, a rat anti-mouse CD3 conjugated to PE and a rat anti-NKp46 conjugated to PE were used, respectively. Before incubation with antibodies, sections were blocked with 5% rat serum in PBS.

### Statistical analysis

Results are reported as means± SD. Statistical significance of differences was assessed by student's *t* test. Anti-tumor activity of the test molecules was assessed by a two-tailed test. The level of significance was set at a P value of less than 0.05.
